# Automated detection of radiolucent foreign body aspiration on chest CT using deep learning

**DOI:** 10.1038/s41746-025-02097-w

**Published:** 2025-11-10

**Authors:** Xiaofan Liu, Zhe Chen, Zhiyong Tang, Xun Yang, Yan Jiang, Dan Zheng, Fangfang Jiang, Fang Ni, Shuang Geng, Qiong Qian, Yan Hao, Junjie Xu, Yin Wang, Mingyuan Zhu, Xiaoqing Wang, Rob M. Ewing, Zehor Belkhatir, Guqin Zhang, Hanxiang Nie, Yi Hu, Weihua Wang, Yihua Wang

**Affiliations:** 1 Department of Pulmonary and Critical Care Medicine, The Central Hospital of Wuhan, Tongji Medical College, Huazhong University of Science and Technology, Wuhan, Hubei 430014 China; 2 Biological Sciences, Faculty of Environmental and Life Sciences, University of Southampton, Southampton SO17 1BJ, UK; 3 Department of Radiology, The Central Hospital of Wuhan, Tongji Medical College, Huazhong University of Science and Technology, Wuhan, Hubei 430014 China; 4 School of Medicine, Jianghan University, Wuhan, Hubei 430056 China; 5 Department of Biostatistics, University of Iowa, Iowa City, IA 52242 USA; 6 Institute for Life Sciences, University of Southampton, Southampton SO17 1BJ, UK; 7 Electronics and Computer Science, Digital Health & Biomedical Engineering Group, University of Southampton, Southampton SO17 1BJ, UK; 8 Department of Respiratory and Critical Care Medicine, Zhongnan Hospital of Wuhan University, Wuhan, Hubei 430071 China; 9 Department of Respiratory and Critical Medicine, Renmin Hospital of Wuhan University, Wuhan, Hubei 430060 China; 10 NIHR Southampton Biomedical Research Centre, University Hospital Southampton, Southampton SO16 6YD, UK

**Keywords:** Respiratory tract diseases, Image processing, Diagnosis, Medical imaging

## Abstract

Radiolucent foreign body aspiration (FBA) remains diagnostically challenging due to its subtle imaging signatures on chest CT scans, often leading to delayed or missed diagnoses. We present a deep learning model integrating MedpSeg, a high-precision airway segmentation method, with a convolutional classifier to detect radiolucent FBA. The model was trained and validated across three independent cohorts, demonstrating consistent performance with accuracies above 90% and balanced recall–precision metrics. In a blinded independent evaluation cohort, the model outperformed expert radiologists in both recall (71.4% *vs*. 35.7%) and F1 score (74.1% *vs*. 52.6%), highlighting its potential to reduce missed cases (false negatives) and support clinical decision-making. This study illustrates the translational potential of artificial intelligence for addressing diagnostically complex and high-risk conditions, offering an effective tool to support radiologists in the assessment of suspected radiolucent foreign body aspiration. Code is available at https://github.com/ZheChen1999/FBA_DL.

## Introduction

Foreign body aspiration (FBA) is a potentially life-threatening condition that disproportionately affects young children and older adults, often resulting in delayed treatment and serious complications when not promptly diagnosed^1–3^^.^ Chest radiography is the primary imaging modality used to identify a foreign body in the lower airway. A retrospective analysis of FBA cases at the Central Hospital of Wuhan, China (2012–2022)^4^, along with a study by Sehgal and colleagues^5^, demonstrated that up to 75% of FBA cases in the adults involve radiolucent foreign bodies. Among these, half of these patients experienced disease duration exceeding 60 days, and two-thirds were misdiagnosed due to the foreign body not being detected early on CT scans^4,6,7^. Symptoms of radiolucent FBA vary widely depending on the location, size, and type of the foreign body, ranging from persistent cough and chest discomfort in adults to acute airway obstruction in infants^8,9^. Radiological imaging plays a critical role in diagnosing FBA. However, radiolucent foreign bodies, which are invisible on conventional radiographs, present a significant diagnostic challenge. Previous studies revealed that approximately 66% of radiolucent foreign body cases were initially misdiagnosed, often as pneumonia^4,10^. This highlights the urgent need for advanced diagnostic methods capable of accurately identifying radiolucent foreign bodies. When radiographic findings are inconclusive, multi-detector computed tomography (MDCT) is clinically indicated due to its superior ability to visualize airway structures and detect subtle pathological changes. However, interpretation remains difficult and diagnostic accuracy is subject to inter-reader variability^11^.

Building on this diagnostic gap, we propose a deep learning (DL)-based framework designed to improve the detection of radiolucent FBAs on chest CT scans^12–14^. Advances in artificial intelligence (AI) and convolutional neural networks (CNNs) have led to notable improvements in airway segmentation and image classification across various thoracic imaging tasks^15^. Although encouraging results have been reported for pediatric FBA detection^16^, identifying radiolucent foreign bodies in adults remains challenging due to their small size, diverse morphology, and resemblance to surrounding tissue structures^17^.

To address these challenges, this study proposes a two-stage deep learning pipeline that integrates high-precision airway segmentation using MedpSeg with multi-view classification via ResNet-18, specifically optimized for radiolucent FBA detection. The model was trained and validated on multiple datasets—including internal modeling, external validation, and independent evaluation cohorts—and its performance was directly compared with expert radiologists. Our model achieved consistent detection with high accuracy and improved diagnostic balance, demonstrating its potential to enhance clinical workflows for suspected radiolucent FBA cases.

## Results

### Patient characteristics

**Fig. 1:**
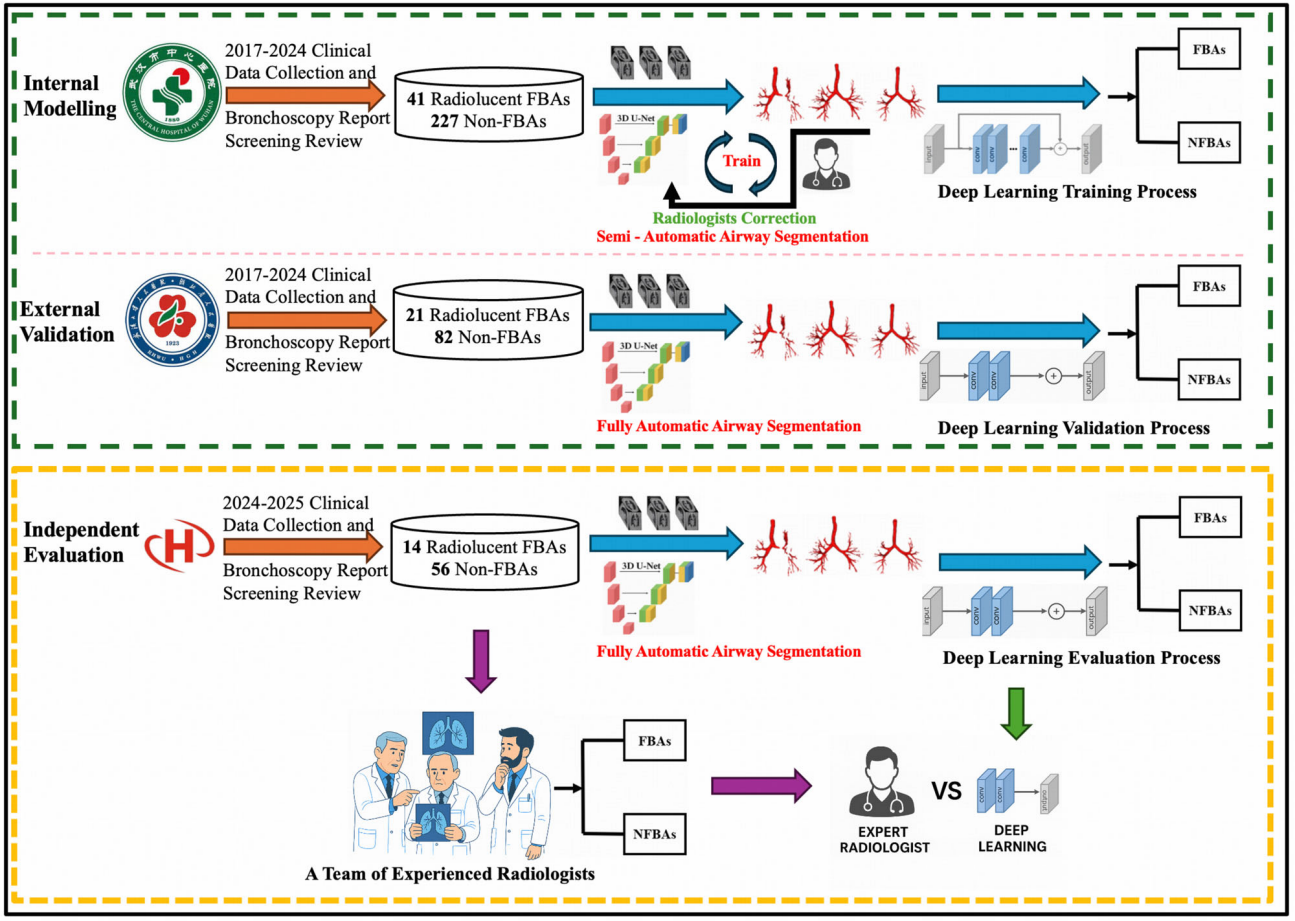
Study design. Workflow illustrating the distribution of participants across the internal modeling, external validation, and independent evaluation cohorts. The independent evaluation cohort included a comparison between three board-certified thoracic radiologists and the deep learning model. FBA Foreign Body Aspiration.

As shown in Supplementary Table 1 and Fig. 1, this study included patients diagnosed with radiolucent FBA from 2017 to 2024 at The Central Hospital of Wuhan and The Renmin Hospital of Wuhan University. A total of 41 radiolucent FBA cases were enrolled in the internal modeling cohort, of whom 26 (63.4%) were male and 15 (36.6%) were female, with a median age of 66 years (interquartile range, IQR 53–77). An additional 21 radiolucent FBA cases were included in the external validation cohort, with a median age of 66 years (IQR 57–70), 13 (61.9%) being male and 8 (38.1%) female. Compared with non-FBA (NFBA) patients, FBA cases had a significantly longer disease course in both the internal modeling and external validation cohorts (60 *vs*. 7 days, *P* < 0.0001) (See Fig.1 for detailed cohort allocation). ICU admission was also more frequent among FBA patients in the internal modeling cohort (22% *vs*. 5%, *P* = 0.0013). Chronic respiratory comorbidities such as chronic obstructive pulmonary disease (COPD, 19.5%), asthma (2.4%) and bronchiectasis (9.8%) were observed in a subset of FBA patients, although without statistically significant differences compared to NFBA patients. Importantly, radiological findings from MDCT demonstrated notable distinctions. In the internal modeling cohort, FBA patients exhibited a significantly higher prevalence of atelectasis (46.3% *vs*. 18.1%, *P* = 0.0002) and a significantly lower prevalence of pleural effusion (0% *vs*. 15.9%, *P* = 0.0125), pulmonary emphysema (9.8% *vs*. 27.4%, *P* = 0.0266) and lung nodules (26.8% *vs*. 42.5%, *P* < 0.0001). In the external validation cohort, pneumonic patches were significantly less common in FBA patients compared to NFBA patients (38.1% *vs*. 96.3%, *P* < 0.0001), whereas the prevalence of tuberculosis (28.6% *vs*. 8.5%, *P* = 0.0236) and airway stenosis (23.8% *vs*. 4.9%, *P* = 0.0163) was significantly higher in the FBA group. The inclusion process for the internal modeling, external validation, and independent evaluation cohorts is illustrated in Fig. 1, providing a clear breakdown of patient sources and exclusion criteria across study sites. This structure underpins the comparative analysis of model generalizability.

Radiological findings from MDCT showed that in the internal modeling cohort, the right middle lobe bronchus (32%) was the most frequent site of radiolucent foreign body, followed by the right upper lobe bronchus (27%). Bone fragments (e.g., chicken, fish, crayfish shell) were the most common type of foreign body (37%), followed by plant materials (27%) (Supplementary Table 2), consistent with earlier studies^4,18–20^.

## 3D airway segmentation

**Fig. 2:**
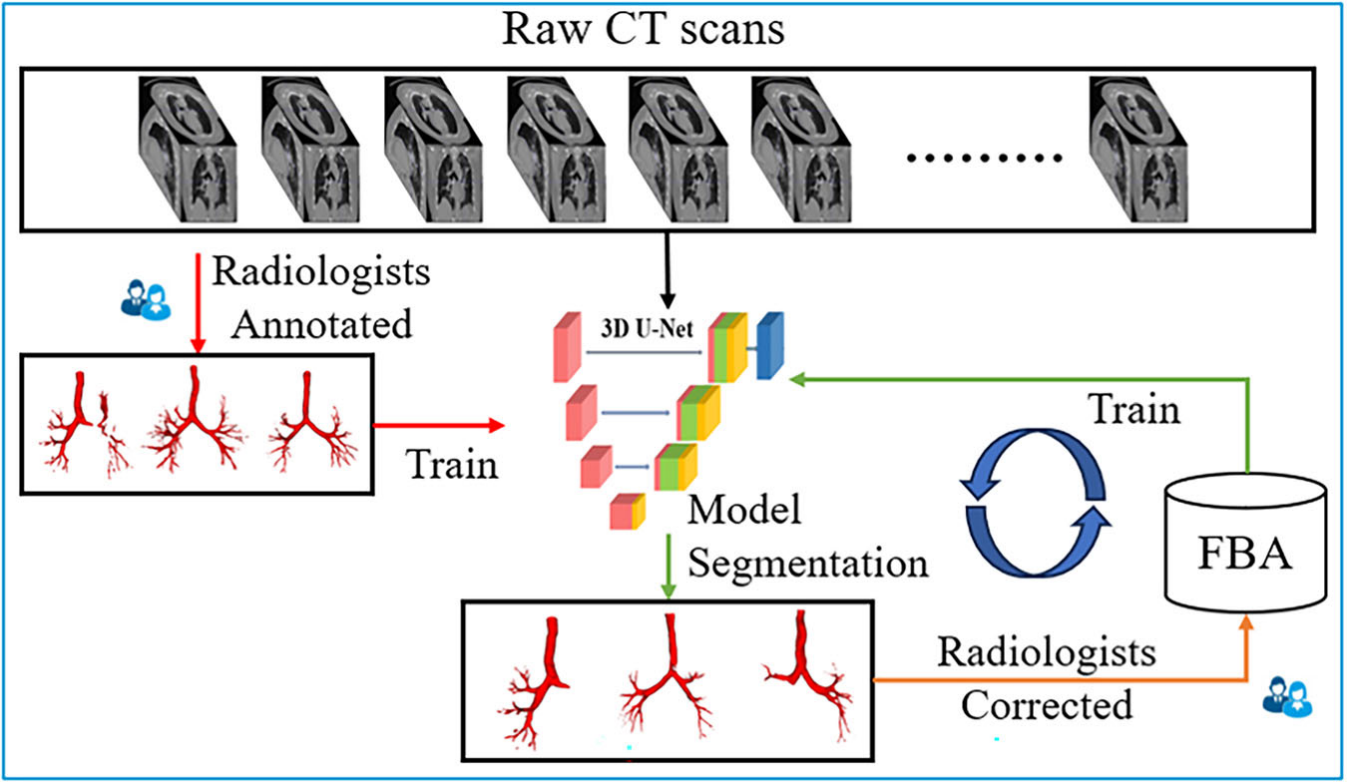
Semi-automated airway labeling process. Semi-automatic airway segmentation combines deep learning and manual correction for efficient, accurate airway CT segmentation. Steps include preprocessing, deep learning model segmentation (e.g., U-Net), manual correction by experts, and iterative training with corrected data. De‑identified CT images are shown with the informed consent requirement waived by the corresponding Ethics Review Committee.

**Fig. 3:**
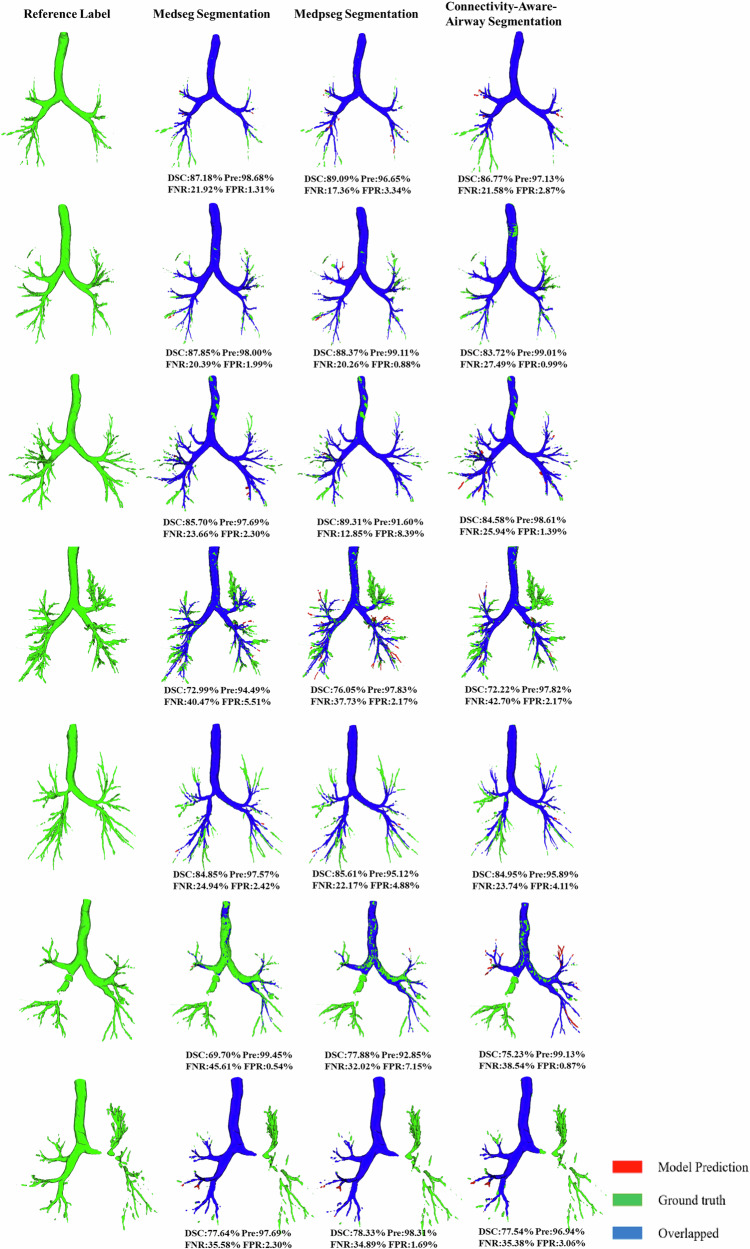
The example airway trees obtained by segmentation using three different methods. The first column shows the gold standard (reference label), while the second, third, and fourth columns depict airway trees reconstructed using the MedSeg, MedpSeg, and AG-UNet (Connectivity-Aware) methods, respectively. In the visualizations, red represents the model prediction, blue indicates the overlap between the model prediction and the gold standard, and green denotes the gold standard.

To ensure anatomical accuracy, a semi-automated labeling workflow was implemented prior to model training, combining deep learning predictions with expert corrections. This process, detailed in Fig. 2, served to refine the airway segmentation ground truth and enhance model robustness against occlusions and branch artifacts. The MedpSeg model, a state-of-the-art deep learning framework, demonstrated outstanding performance in airway segmentation, consistently outperforming MedSeg and AG-UNet, across key evaluation metrics. In the internal modeling cohort, MedpSeg achieved the highest dice similarity coefficient (DSC) of 87.48% and the lowest average symmetric surface distance (ASSD) of 0.71 mm, indicating superior segmentation accuracy and boundary precision. In the external validation cohort, the model maintained robust performance with a DSC of 86.58% and an ASSD of 0.75 mm, further validating its generalizability. Compared to MedSeg and AG-UNet, MedpSeg exhibited superior precision in capturing intricate airway structures, particularly in cases involving partial obstructions. As shown in Table 1 and Fig. 3, the segmentation results from MedpSeg aligned most closely with the gold standard (ground truth), demonstrating minimal false negatives and false positives. These findings underscore MedpSeg’s potential to improve airway segmentation accuracy, offering a reliable basis for subsequent foreign body detection and localization. The model’s consistent performance across internal and external datasets highlights its robustness and applicability in diverse clinical settings. Once trained, MedpSeg performed segmentation without any human intervention (Fig. 1).

**Table 1:** Segmentation performance comparison across methods in the internal modeling and external validation cohorts

Internal Modeling	DSC (%)	VOE (%)	RVD (%)	ASSD (mm)	Pre (%)	FNR (%)	FPR (%)	MIOU (%)
MedSeg	86.54	20.43	19.37	0.76	99.70	20.52	1.29	0.854
MedpSeg	87.48	18.23	18.75	0.71	99.89	20.15	1.22	0.897
AG-Unet	85.24	21.37	20.13	0.82	99.57	21.45	1.35	0.793

External Validation	DSC (%)	VOE (%)	RVD (%)	ASSD (mm)	Pre (%)	FNR (%)	FPR (%)	MIOU (%)
MedSeg	85.35	21.25	20.58	0.81	99.58	21.23	1.45	0.837
MedpSeg	86.58	18.75	19.28	0.75	99.81	21.05	1.37	0.878
AG-Unet	84.57	22.15	21.58	0.85	99.33	22.13	1.58	0.775

*DSC* dice similarity coefficient, *VOE* volumetric overlap error, *RVD* relative volume difference, *ASSD* average symmetric surface distance, *Pre* precision, *FNR* false negative rate, *FPR* false positive rate, *MIOU* mean intersection over union.

### A deep learning method to detect radiolucent FBA on chest CT scans

We then explored various classification backbones, including ResNet-18, EfficientNet-B0, DenseNet-121, ViT-B/16, and Swin-T (tiny). As shown in Supplementary Table S3, ResNet-18 achieved the best balance between predictive performance and computational efficiency, making it particularly suitable for real-time clinical deployment. While some transformer-based models (e.g., ViT-B/16, Swin-T) demonstrated competitive recall, they tended to underperform in precision and overall F1 score, likely due to their higher parameter complexity and sensitivity to smaller datasets. These results suggest that ResNet-18 offers a favorable trade-off between model complexity and performance in the context of radiolucent FBA detection.

**Fig. 4:**
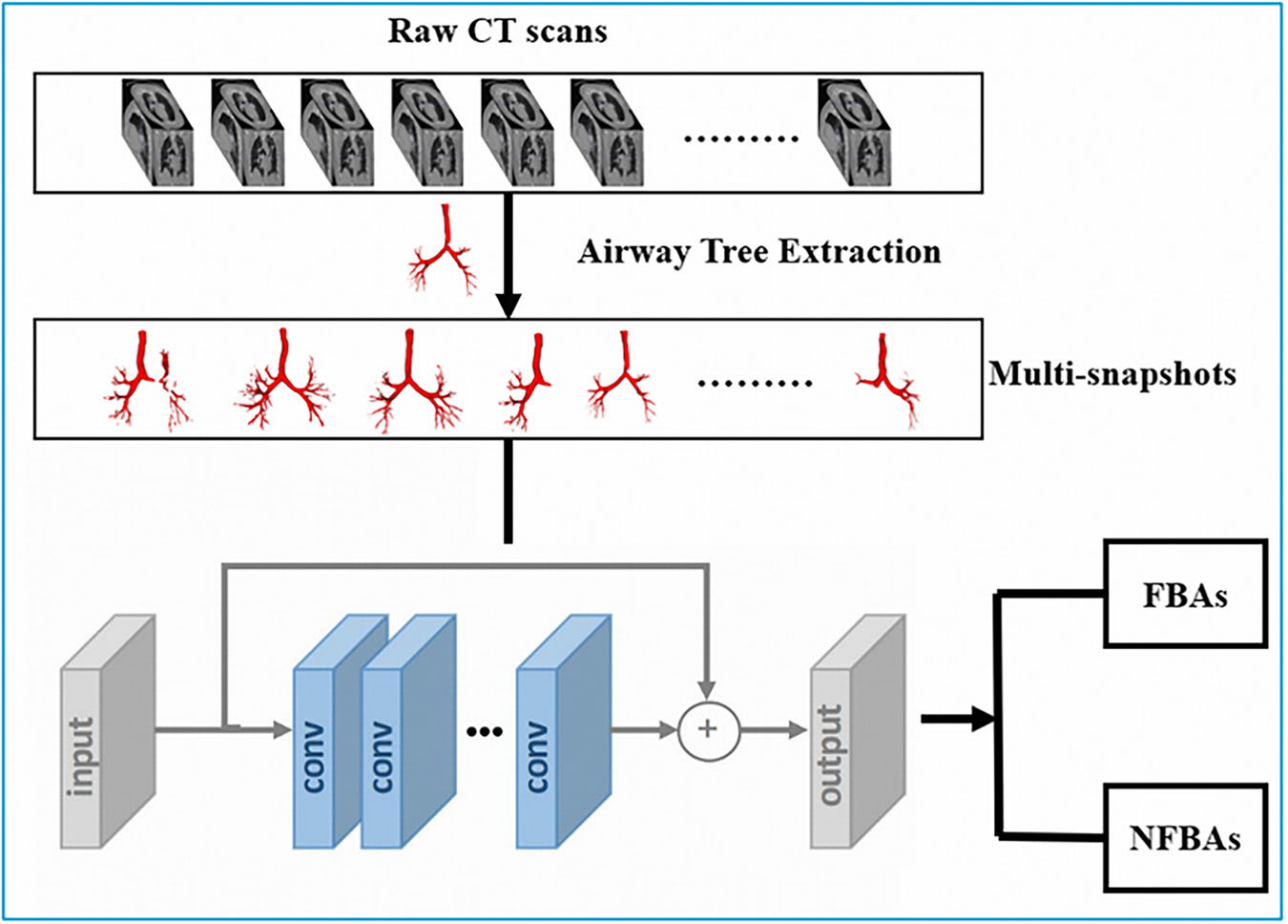
The proposed workflow of the multi-view-based image classification for foreign body aspiration detection*.* In brief, the CT images undergo preprocessing and airway tree extraction to generate 3D airway models. Multi-snapshots of these models are taken from different angles. These snapshots are then processed using a convolutional neural network (CNN) architecture, which includes convolution, max pooling, and fully connected layers. Finally, the processed images are classified into two categories: FBA (foreign body aspiration) and NFBA (non-foreign body aspiration). De-identified CT images, identical to those presented in Fig. 2, are shown with the informed consent requirement waived by the corresponding Ethics Review Committee.

To enable accurate detection of radiolucent FBA, we implemented a two-stage deep learning framework combining anatomical feature extraction with multi-view image classification. As illustrated in Fig. 4, segmented 3D airway structures are captured from multiple angles to generate a set of snapshot views. These multi-angle images are fed into a ResNet-18 convolutional neural network, which includes convolutional, pooling, and fully connected layers, fine-tuned for binary classification between FBA and NFBA. This multi-view strategy improves the model’s ability to localize subtle, non-radiopaque foreign bodies that may be overlooked in standard slice-wise analysis.

**Fig. 5:**
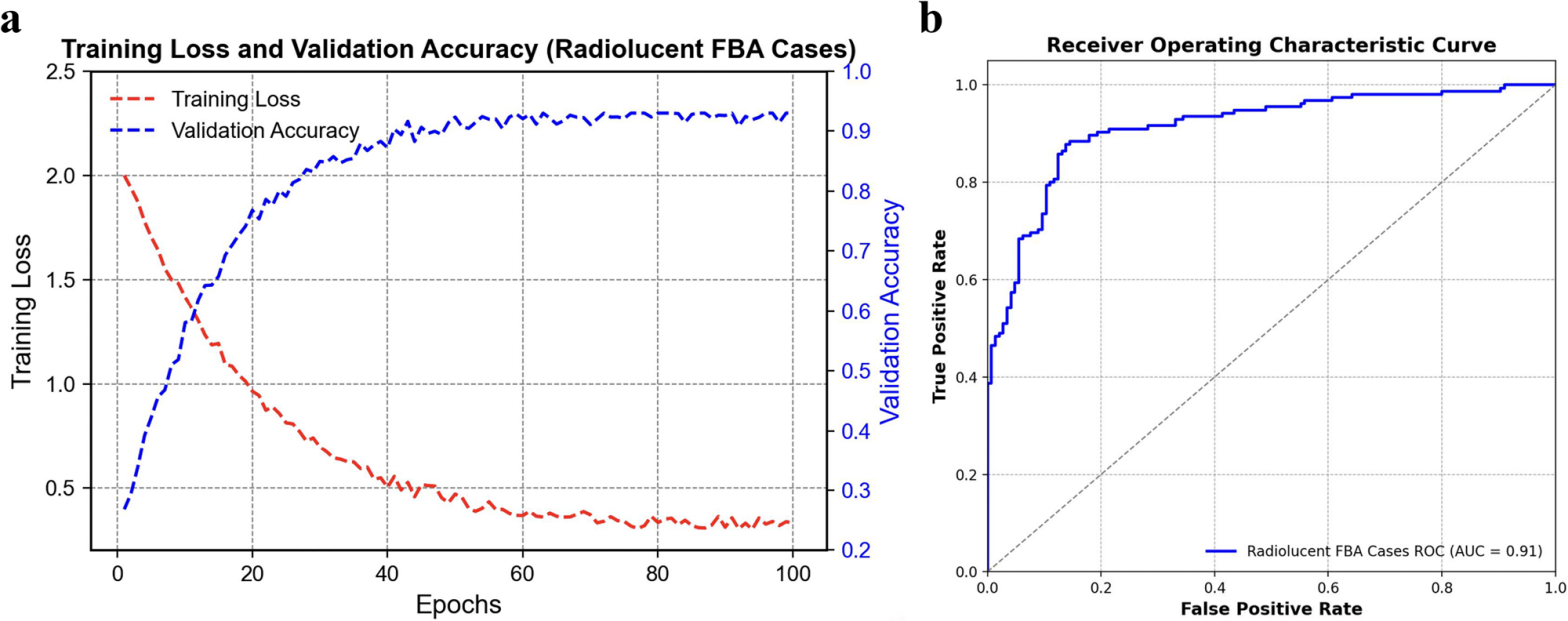
A deep learning model to detect radiolucent foreign body aspiration (FBA) in chest CT scans. **a** The training loss and validation accuracy of a Resnet-18 model over 100 epochs for radiolucent FBA cases. The training loss is indicated in red and the validation accuracy in blue. **b** The receiver operating characteristic curve for the internal modeling to detect radiolucent FBA cases. AUC area under the curve; ROC receiver operating characteristic.

The training loss and validation accuracy trends for the ResNet-18 model over 100 epochs were examined in the radiolucent FBA cases (Fig. 5a). The rapid decline in training loss (red line) during the initial epochs indicates effective learning, with errors stabilizing at a low level. Simultaneously, the validation accuracy (blue line) steadily increases and stabilizes near 0.9, reflecting strong generalization to unseen data. Minor oscillations in validation accuracy suggest potential overfitting or inconsistencies in the validation dataset, warranting further optimization through regularization techniques or hyperparameter tuning to improve robustness. We then checked the Receiver Operating Characteristic (ROC) curve for the model to detect radiolucent FBA cases. The area under the ROC curve (AUC) for radiolucent FBA detection was 0.91 (95% confidence interval, CI: 0.86–0.95) for the internal modeling cohort, 0.88 (95% CI: 0.82–0.94) for the external validation cohort, and 0.89 (95% CI: 0.83–0.96) for the independent evaluation cohort. Pairwise comparisons using DeLong’s test revealed no statistically significant differences in AUCs between the cohorts (internal modeling *vs*. external validation: *P* = 0.31; internal modeling *vs*. independent evaluation: *P* = 0.45; external validation *vs*. independent evaluation: *P* = 0.67), indicating consistent and robust diagnostic performance in detecting radiolucent FBA cases across datasets (Fig. 5b).

Model performance was also evaluated in both the internal modeling and external validation cohorts. As shown in Table 2, the model achieved an accuracy of 94.4%, recall of 78.0%, precision of 84.2%, and F1 score of 81.0% in the internal cohort. In the external validation cohort, accuracy was 90.3%, recall 76.2%, precision 76.2%, and F1 score 76.2%.

**Table 2:** Performance of the deep learning model for radiolucent FBA

Radiolucent FBA cohort	TP	FN	TN	FP	Accuracy	Precision	Recall	F1 Score
**Internal Modeling (*n*** = **268)**	32 (11.9%)	9 (3.4%)	221 (82.5%)	6 (2.2%)	94.4%	84.2%	78.0%	81.0%
**External Validation (*n*** = **103)**	16 (15.5%)	5 (4.9%)	77 (74.8%)	5 (4.9%)	90.3%	76.2%	76.2%	76.2%

Internal modeling and external validation cohorts.

Data are *n* (%). *FBA* foreign body aspiration, *TP* true positive, *FP* false positive, *TN* true negative, *FN* false negative.

Note: Values in parentheses represent the percentage relative to the full cohort. Percentages for TP/FN/TN/FP are calculated with a denominator = *n* (FBA + NFBA cases).

To further evaluate generalization, we performed an age-based subgroup analysis within the independent evaluation cohort. Patients were divided into two groups: individuals under 40 years old and those aged 40 years or older. Among individuals under 40 years old (*n* = 12), the model achieved an accuracy of 83.3%, precision of 75.0%, recall of 75.0%, and F1 score of 75.0%. For those aged 40 years or above (*n* = 58), the accuracy was 91.4%, with precision, recall, and F1 score of 77.8%, 70.0%, and 73.7%, respectively (Supplementary Table S4). Fisher’s exact tests for recall, precision, and accuracy revealed no statistically significant differences between the two age groups (all *P* > 0.5), suggesting that the model maintains consistent classification performance across age strata. These results demonstrate that the model generalizes well across different ages.

### An ablation study to evaluate the contributions of each pipeline component

For this purpose, we conducted a comprehensive ablation study using the independent evaluation cohort (*n* = 70). Beginning with a baseline model trained solely on raw axial CT slices, we sequentially incorporated segmentation masks, reduced-view projections, and data augmentation. As shown in Supplementary Table S5, the baseline model yielded an accuracy of 72.3% and an F1 score of 44.6%. The inclusion of segmentation masks modestly improved performance (accuracy: 75.8%; F1 score: 57.3%), and adopting a reduced multi-view strategy (six projection views) further enhanced performance to 79.6% accuracy and a 59.6% F1 score. The addition of data augmentation contributed substantial gains in generalizability, resulting in 85.4% accuracy and a 65.9% F1 score. The complete pipeline—incorporating segmentation, augmentation, and a full set of 12 projection views—achieved the highest overall performance, with an accuracy of 90.0% and an F1 score of 74.1%. These findings underscore the synergistic benefits of structural modeling, enhanced spatial context, and data diversity in detecting subtle features associated with radiolucent FBA.

### Evaluating the performance of deep learning vs. expert radiologists

Finally, we assembled an independent evaluation cohort from The Zhongnan Hospital of Wuhan University, consisting of 14 bronchoscopy-confirmed radiolucent FBA cases and 56 NFBA controls (Fig. 1). As detailed in Supplementary Table 6, among the radiolucent FBA cases, 8 (57.2%) were male and 6 (42.8%) were female, with a median age of 56 years (IQR 38–74). CT scans were independently reviewed by three board-certified thoracic radiologists (each with over 10 years of clinical experience) blinded to bronchoscopy findings; any discrepancies were resolved by consensus (Fig. 1). Table 3 presents the comparative performance metrics of the deep learning model and the expert radiologists on this independent evaluation cohort. The deep learning model achieved an accuracy of 90.0%, with a precision of 76.9%, a recall of 71.4%, and an F1 score of 74.1%. In comparison, expert radiologists demonstrated a perfect precision of 100% but a lower recall of 35.7%, resulting in an overall accuracy of 87.1% and an F1 score of 52.6%. Notably, the deep learning model outperformed experienced radiologists in both recall (71.4% vs. 35.7%; *P* < 0.05) and F1 score (74.1% *vs*. 52.6%; *P* < 0.05). The F1 score, as the harmonic mean of precision and recall, provides a balanced metric that is especially informative in the context of imbalanced datasets. These results highlight the model’s potential to reduce missed cases (false negatives) while maintaining acceptable precision, thereby supporting clinical decision-making.

**Table 3:** Performance comparison between the deep learning model and expert radiologists in the independent evaluation cohort for radiolucent FBA cases

Radiolucent FBA cohort (*n* = 70)	TP	FN	TN	FP	Accuracy	Precision	Recall	F1 Score
**Deep Learning Model**	10 (14.3%)	4 (5.7%)	53 (75.7%)	3 (4.3%)	90.0%	76.9%	71.4%	74.1%
**Expert Radiologists**	5 (7.1%)	9 (12.9%)	56 (80%)	0 (0%)	87.1%	100%	35.7%	52.6%

Data are *n* (%).

Values in parentheses represent the percentage relative to the full evaluation cohort (*N* = 70; 14 FBA and 56 NFBA). Percentages for TP and FN are calculated with denominator = 14 (FBA cases), and for TN and FP with denominator = 56 (NFBA cases).

*FBA* foreign body aspiration, *TP* true positive, *FP* false positive, *TN* true negative, *FN* false negative.

*P* values with McNemar’s test.

## Discussion

FBA poses significant clinical challenges, often leading to prolonged disease courses and high rates of misdiagnosis. One of the primary difficulties in diagnosing FBA is that many foreign bodies are radiolucent, making them nearly invisible on routine imaging methods, including X-rays and CT scans. In addition, patients may lack a clear recollection of an aspiration event—such as choking or coughing while eating—further complicating the diagnosis. This study demonstrates that deep learning models can effectively address these challenges, particularly in identifying radiolucent FBAs, by leveraging advanced CT imaging analysis.

Accurate airway segmentation is crucial for identifying FBA cases. In this study, airway segmentation during training was performed using the MedpSeg deep learning model, with radiologists-guided corrections incorporated to ensure accurate and reliable airway mapping, achieving a DSC of 87.48%. This approach achieved higher segmentation accuracy compared to conventional methods like region growing or wave propagation, which typically achieve DSC values around 70–80%^21^. Compared to benchmarks, such as U-Net variants used in competitions like EXACT'09, our approach exhibited greater robustness, especially in cases involving airway obstruction. Although previous studies validated the applicability of U-Net and similar architectures, their dependence on manual preprocessing or post-segmentation adjustments limited their scalability^22–24^. Performance on a 10-case hold-out set, unseen during iterative refinement, confirmed the absence of overfitting or feedback-related inflation. By integrating manual corrections into iterative training, we reduced false positives and improved generalizability.

In detecting radiolucent FBAs, the ResNet-18-based classification model achieved excellent performance across three distinct datasets. Accuracy ranged from 90.0% to 94.4%, with precision between 76.2% and 84.2%, and recall between 76.2% and 78.0%. These results are especially significant when compared to expert radiologists’ performance in the independent evaluation cohort: although human readers achieved perfect precision (100%), their recall dropped markedly to 35.7%, indicating a high false-negative rate. The model, in contrast, offered a more balanced trade-off between recall and precision, with a higher F1 score (74.1% vs. 52.6%), highlighting its value as a second-reader tool to reduce missed diagnoses and to prioritize appropriate bronchoscopic evaluation.

Previous studies have explored a range of strategies for detecting foreign body aspiration, including radiographic interpretation, rule-based diagnostic models, and conventional machine learning methods^25–27^. However, these methods often lacked specificity to radiolucent FBA, were limited to radiography, or were validated only on small, homogeneous datasets. For example, retrospective studies have shown that up to 66% of radiolucent FBAs are initially misdiagnosed as pneumonia or asthma, due to the subtlety of CT findings and absence of radiopaque markers^4^. Traditional machine learning approaches, such as support vector machines or radiomics-based classifiers, have reported moderate performance (AUC ~ 0.75–0.80) in small datasets but lacked validation across independent cohorts. More recently, Truong and colleagues applied deep learning to pediatric chest X-rays for FBA detection^12^, achieving an AUC of 0.88, but their method was not applicable to radiolucent cases or CT-based workflows. Similarly, airway segmentation frameworks by Charbonnier et al. and Garcia-Uceda Juarez et al. focused on anatomical reconstruction but did not address FBA detection directly^22,28^. Our study addresses this gap by focusing specifically on radiolucent FBA and validating across three distinct institutional cohorts using CT imaging.

Despite the encouraging results, several limitations must be acknowledged. First, the retrospective design of this study introduces potential selection bias and the overall sample size remains relatively limited. To help mitigate this, we incorporated data from three independent hospitals in Wuhan (China), each with different radiology departments and CT scanner models (UCT780 64-row, Philips Brilliance iCT, Canon Aquilion One, UCT780 80-row). Although geographically close, this setup introduces a degree of institutional heterogeneity, which helps approximate certain aspects of multi-center validation. Furthermore, these hospitals recruited patients from across central China and beyond, encompassing a range of population characteristics (*e.g*., age distribution, diet, and comorbidity profiles). Second, although CT imaging provides excellent spatial resolution for airway assessment, its relatively high radiation dose limits widespread application, particularly for screening or serial follow-up in pediatric populations. Our model is therefore intended for targeted use in cases with clinical suspicion following inconclusive X-ray findings. Future work will explore integration with low-dose CT protocols and assess the cost-effectiveness and clinical impact of this approach in triage workflows. Third, radiolucent FBAs represent a minority class in real-world clinical datasets, complicating model optimization. To improve this, we adopted focal loss, class-balanced mini-batching, and data augmentation strategies during training. Additional techniques such as synthetic oversampling or semi-supervised learning could further improve model sensitivity^29,30^. Fourth, the model demonstrated higher recall than expert radiologists but slightly lower precision, raising the possibility of more false-positive cases. However, it is designed as a second-reader or triage tool—rather than a diagnostic replacement—and may assist clinicians by highlighting subtle airway-localized changes suggestive of radiolucent FBA, especially when CT findings are inconclusive. Lastly, the model currently relies solely on imaging features. Incorporating additional clinical metadata—such as symptom duration, aspiration history, and comorbidities—into a multimodal AI framework could further enhance diagnostic accuracy and clinical decision support.

**Fig. 6:**
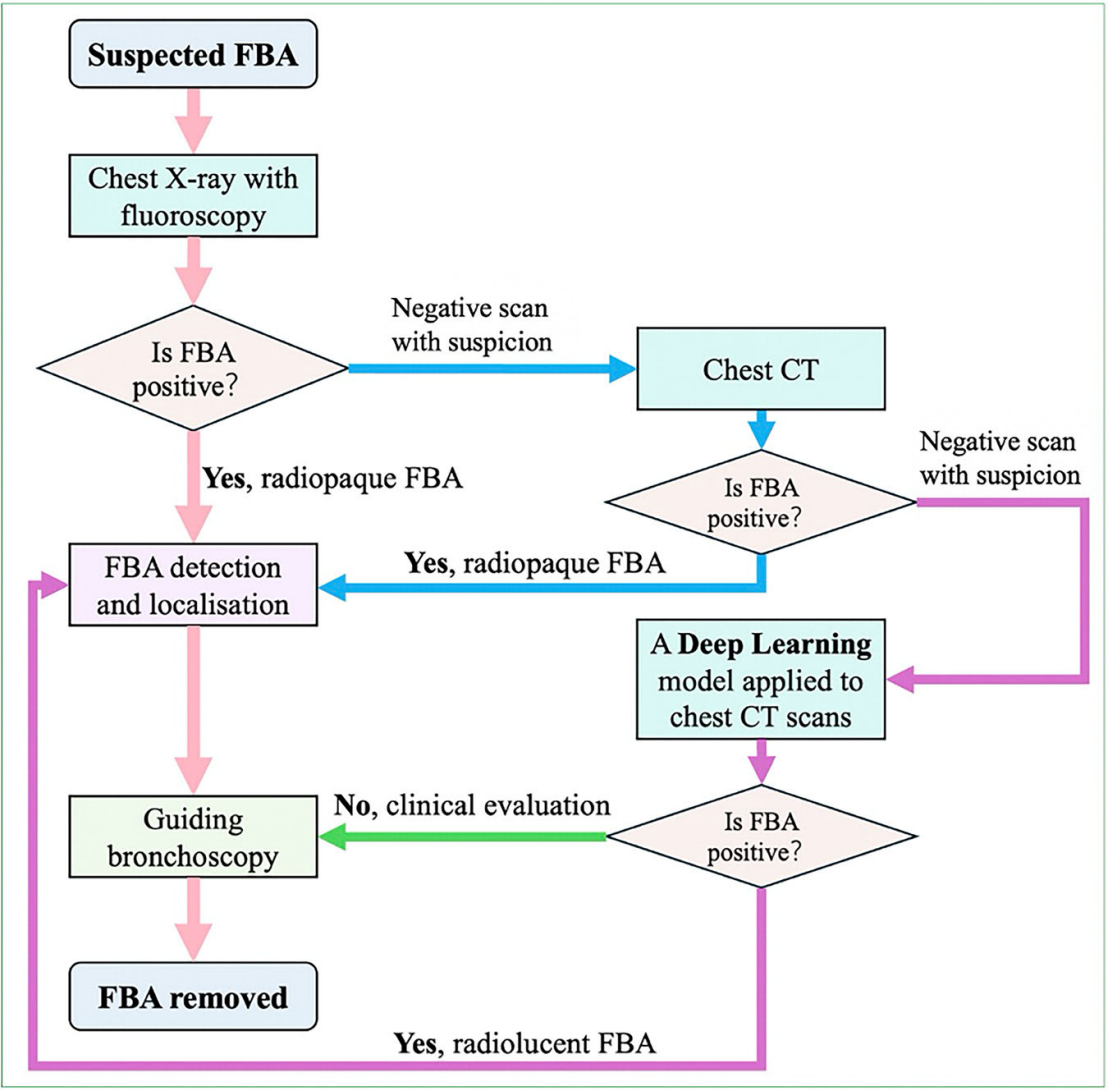
The proposed diagnostic workflow for detecting foreign body aspiration (FBA). The stepwise approach begins with chest X-rays to identify radiopaque FBA. For suspected radiolucent FBA, chest CT scans are recommended. If imaging is inconclusive, applying a deep learning model to CT scans enhances the detection of subtle abnormalities. This workflow guides bronchoscopy for precise removal while minimizing complications.

The proposed diagnostic workflow (Fig. 6) for FBA detection and localization outlines a stepwise, evidence-based approach. Initial assessments using chest X-rays provide a rapid, non-invasive method to identify radiopaque FBAs. For suspected radiolucent FBA cases, further evaluation with chest CT scans is recommended. When conventional imaging methods fail to detect abnormalities, applying a deep learning model to CT scans enhances detection capabilities, particularly for subtle abnormalities that might escape manual interpretation. This approach effectively guides bronchoscopy for precise removal, minimizing complications such as airway damage or prolonged obstruction.

This study highlights the clinical value and transformative potential of deep learning in the diagnosis of radiolucent FBA. By integrating accurate airway segmentation with multi-view CT classification, the proposed model achieved high accuracy, generalizability across internal modeling, external validation, and independent evaluation cohorts, and demonstrated superior recall and F1 score compared to experienced radiologists, with slightly lower precision. These findings suggest that deep learning-based systems can effectively complement clinical workflows by reducing missed cases, maintaining acceptable false-positive rates, and supporting more targeted bronchoscopic interventions. Prospective, multi-center studies with larger and more diverse populations are necessary to improve model robustness and reduce site-specific or demographic bias^31^.

## Methods

### Patient cohorts and data acquisition

As shown in Fig. 1, this study utilized a multi-source, multi-center dataset encompassing both publicly available and clinically acquired thoracic CT scans. Our datasets reflect real-world heterogeneity in demographics and imaging conditions, enhancing the external validity of our findings.

### Airway Tree Modeling 2022 (ATM22) challenge

Comprises 500 computed tomography (CT) scans, with 300 allocated for training, 50 for external validation, and 150 for testing, which were sourced from the publicly available LIDC-IDRI dataset and the Shanghai Chest Hospital^33,34^. Initial preprocessing of the CT images involved employing robust deep-learning models and an ensemble technique to generate preliminary segmentation results. Subsequently, three experienced radiologists, possessing a cumulative expertise exceeding ten years, meticulously outlined and cross-verified these results to derive the final refined airway tree structure.

### The internal modeling dataset

Was derived from patients screened at The Central Hospital of Wuhan. It included data from both FBA (Foreign Body Aspiration) patients and a randomly selected cohort of NFBA (Non-FBA) patients. For FBA patients, an initial pool of 81 cases was identified through clinical records and bronchoscopy reports. From this pool, 23 cases were excluded due to missing or incomplete clinical data, leaving a total of 58 patients in the final dataset. Among these, 17 cases were classified as radiopaque, where foreign bodies were visible on CT scans, while the remaining 41 cases were categorized as radiolucent, where no visible foreign bodies were detected on CT images but were confirmed through bronchoscopy. For the NFBA cohort, a total of 260 hospitalized patients were randomly selected from the same hospital during the study period. After excluding 33 cases with incomplete CT imaging or missing diagnostic records, 227 patients were retained in the final dataset.

### The external validation dataset

Was collected from The Renmin Hospital of Wuhan University to test the generalizability of the model. Data collection followed a similar process for the internal modeling dataset. For FBA patients, 49 cases were initially screened based on clinical data and bronchoscopy reports. After excluding 19 cases with incomplete clinical records, a total of 30 cases were included in the final dataset. Among these, 9 cases were classified as radiopaque, and 21 cases were classified as radiolucent. For NFBA patients, 128 hospitalized patients were randomly selected during the same timeframe. After excluding 27 cases with missing or incomplete imaging or diagnostic data, 82 patients were retained.

### The independent evaluation dataset

(The Zhongnan Hospital of Wuhan University) was compiled to assess the real-world generalizability of the proposed model in a clinical setting. A total of 70 patients were retrospectively included based on bronchoscopy-confirmed diagnoses and availability of high-quality CT imaging. Of 18 FBA cases reviewed at the Zhongnan Hospital of Wuhan University, 14 were classified as radiolucent and included in the independent evaluation cohort. The remaining 4 radiopaque cases were excluded prior to preprocessing. The NFBA cohort comprised 56 hospitalized patients, randomly selected during the same time period. These patients were confirmed to have no evidence of foreign body aspiration based on clinical history, imaging, and bronchoscopy when applicable. This independent dataset provided a robust platform to evaluate the model’s diagnostic performance in a real-world, heterogenous clinical environment, and was used to benchmark the model’s performance against expert radiologist interpretation.

### Geographic and clinical diversity

Collectively, these datasets encompass diverse patient populations from three independent tertiary centers within Wuhan, China—each serving different districts and referral patterns. This regional diversity enhances the robustness of model evaluation and supports broader generalizability. Importantly, while all data were from Chinese institutions, the combination of multi-institutional sourcing, varied scanner protocols, and heterogenous inpatient demographics enhances the translational relevance of our findings.

### CT image preprocessing

To ensure consistency and reproducibility across imaging data obtained from multiple institutions and CT scanner vendors, all chest CT scans were subjected to a standardized preprocessing pipeline prior to airway segmentation and classification. First, volumetric data were resampled to an isotropic voxel spacing of 1.0 × 1.0 × 1.0 mm³ using trilinear interpolation to harmonize spatial resolution and enable uniform processing of three-dimensional anatomical structures. Voxel intensities were clipped to a fixed Hounsfield Unit (HU) range of [–1000, +400 HU], capturing the attenuation characteristics of air-filled airways, pulmonary parenchyma, and soft tissue while excluding high-density bone and metal artifacts. The clipped values were then normalized to the [0, 1] range using min–max scaling to facilitate numerical stability during model optimization. For inputs to the classification network, an additional Z-score normalization step was applied to 2D airway projection images to match the distribution of pretrained ImageNet features and improve downstream feature alignment during transfer learning. To ensure consistent input dimensions for the 3D segmentation network, all CT volumes were either centrally cropped or zero-padded to a standardized shape of 128 × 128 × 128 voxels, empirically determined to capture the full extent of the tracheobronchial tree while balancing computational load. In cases with large fields of view, anatomical centering based on the airway centroid was applied to preserve relevant structures. Finally, a Gaussian smoothing filter (σ = 1.0) was applied to each CT volume. This denoising step improved segmentation boundary clarity and supported accurate mesh-based airway surface reconstruction used for subsequent classification.

### Data augmentation

To enhance model generalizability across diverse anatomical presentations and imaging conditions, as well as to mitigate overfitting due to moderate class imbalance, a comprehensive data augmentation strategy was applied during training of both the airway segmentation and classification networks. For 3D segmentation tasks, online augmentations included random flipping along all three spatial axes, small-angle rotations (±15°), and elastic deformations to simulate realistic variations in airway curvature and subsegmental branching. Additionally, random cropping, Gaussian noise injection (mean = 0, standard deviation = 0.02), and brightness/contrast perturbations (±15%) were used to mimic inter-scanner variability and noise introduced by low-dose protocols. For the classification network, which operates on 2D rendered views of segmented airway surfaces, augmentations were applied at the image level. These included random affine transformations (rotation, translation, and zoom within ±10%), circular occlusion masking to simulate segmentation dropouts or partial obstruction, and color jittering to account for rendering variations. Furthermore, view dropout was implemented by randomly omitting one to two views out of the 12 total per subject, forcing the model to rely on incomplete visual context and improving robustness to partial input. All augmentations were applied dynamically during mini-batch generation using a fixed random seed for reproducibility. The augmentation parameters were empirically optimized based on performance on a held-out validation set, and all transformations were constrained to maintain anatomical plausibility of airway geometry.

### Class imbalance mitigation

Radiolucent FBA represents a rare but clinically significant diagnostic challenge, resulting in a marked class imbalance between positive (radiolucent FBA) and negative (non-FBA) cases. To mitigate this imbalance and support robust model training, we implemented a suite of complementary strategies aimed at improving sensitivity while preserving specificity.

First, we employed stratified mini-batch sampling to enforce a 1:1 ratio of radiolucent FBA to non-FBA cases in each training batch. This sampling approach ensured consistent exposure to the minority class and stabilized learning dynamics across epochs, reducing the risk of the model converging toward a trivial majority-class solution. Second, to further address class imbalance during optimization, we adopted focal loss as the primary objective function^35^. Focal loss emphasizes hard-to-classify examples by down-weighting well-classified instances. Specifically, we set the focusing parameter *γ* to 2.0 and the class weight *α* to 0.25 for positive samples. This formulation enabled the model to concentrate learning on subtle, ambiguous cases typical of radiolucent FBA. The Focal Loss ${{\mathcal{L}}}_{{\rm{Focal}}}$ is defined as:

1 $${{\mathcal{L}}}_{{\rm{Focal}}}=-{\alpha }_{t}{(1-{p}_{t})}^{\gamma }\log {\rm{}}({p}_{t})$$

where ${p}_{t}$ is the predicted probability for the ground truth class, ${a}_{t}\in [0,1]$ is a weighting factor to balance positive and negative voxels, $\gamma \ge 0$ is the focusing parameter that adjusts the rate at which easy examples are down-weighted.

### Airway 3D reconstruction

For airway 3D reconstruction, a combination of publicly available datasets and hospital-derived CT scans was used. The primary dataset, sourced from the Airway Tree Modelling 2022 (ATM22) challenge, included 500 CT scans (300 for training, 50 for external validation, and 150 for testing) derived from the LIDC-IDRI dataset and the Shanghai Chest Hospital^33,34^. We adopted the MedpSeg model, a state-of-the-art deep learning framework, for airway segmentation^36^. The MedpSeg model was designed to address the inherent anatomical challenges of airway segmentation, including fine branching structures, large inter-patient variability, and class imbalance between airway and background voxels. The architecture followed an encoder–decoder configuration with skip connections and included residual blocks to facilitate gradient flow and channel-wise attention mechanisms to improve feature selectivity in decoder layers. Each encoder block consisted of two 3D convolutional layers (kernel size 3 × 3 × 3), batch normalization, and ReLU activation, followed by 3D max pooling (2 × 2 × 2). Decoder blocks employed transposed convolutions for upsampling, combined with symmetric encoder features. A final 1 × 1 × 1 convolution followed by sigmoid activation produced binary airway masks.

The initial segmentation outputs were reviewed and refined by radiologists, who corrected missing airway branches or removed erroneous segmentations (a semi-automated workflow; Fig. 2). Segmentation training involved a three-round iterative refinement workflow with radiologist feedback. In Iteration 1, the pretrained MedpSeg model (ATM22) was applied to the 60 internal hospital CTs. The outputs were reviewed and manually corrected by three board-certified thoracic radiologists (≥10 years experience), addressing missing branches, false positives, and segmentation discontinuities. In Iteration 2, the model was retrained on these corrected masks and applied to a new subset of scans; approximately 20% of outputs required further correction. In Iteration 3, final retraining was performed on the cumulative corrected dataset, after which <10% of cases required minimal edits (average correction time 8–12 min vs. 25–30 min for full manual labeling). This semi-automated human-in-the-loop workflow significantly reduced annotation burden while preserving anatomical fidelity. Notably, no manual correction or retraining was performed during model inference in any of the validation or test phases, ensuring full automation for downstream clinical application.

This iterative process progressively improved model accuracy while significantly reducing manual workload. Once trained, MedpSeg performed segmentation without any human intervention. No manual correction or retraining is required for external use. During training, approximately 40% of the cases initially required manual correction to fix missing branches or reduce false positives. After several rounds of retraining and refinement, the manual intervention rate decreased to below 10%. Each correction took 8–12 min on average, compared to 25–30 min for full manual annotation, representing a substantial reduction in workload. No manual correction was used during model inference in the validation or evaluation phases.

Segmentation accuracy was quantitatively evaluated using a comprehensive set of metrics: DSC, Average Symmetric Surface Distance (ASSD), Volumetric Overlap Error (VOE), Relative Volume Difference (RVD), and Mean Intersection over Union (mIoU). On a held-out internal test subset excluded from all training phases, MedpSeg achieved DSC = 86.8% and ASSD = 0.73 mm, with comparable performance on the external validation cohort, indicating excellent generalization. The final segmentation outputs were converted to triangulated surface meshes for 3D airway rendering and multi-view projection, serving as input to the classification pipeline.

### Foreign body aspiration identification based on deep learning

As shown in Fig. 3, the workflow for multi-view-based FBA classification involves several key steps. Initially, raw CT images undergo preprocessing and airway tree extraction, producing detailed 3D airway models. These models are then captured from multiple viewpoints to generate a series of 2D snapshots, which serve as input for a convolutional neural network (CNN) classifier.

To facilitate reproducibility, all critical preprocessing and rendering parameters have been made publicly available via our GitHub repository. CT volumes were clipped to a Hounsfield Unit (HU) range of –1000 to +400, resampled to isotropic voxel dimensions of 1.0 mm³, and intensity-normalized using min-max scaling. For airway rendering, 12 uniformly spaced snapshot views were generated across the 3D airway surface using a virtual camera radius of 150 mm. Each image was rendered at a resolution of 224 × 224 pixels and subsequently fed into a ResNet-18 classifier.

Each rendered 2D projection was passed through a ResNet-18 backbone, pre-trained on ImageNet and fine-tuned for binary classification. The network consists of four residual convolutional blocks, batch normalization, ReLU activations, and global average pooling, followed by a fully connected classification head comprising a 256-unit dense layer, dropout (*p* = 0.5), and SoftMax output. Final patient-level predictions were obtained by averaging SoftMax probabilities across all 12 views.

Model optimization was performed using the Adam optimizer with an initial learning rate of 1 × 10⁻⁴, reduced by a factor of 0.1 if validation loss plateaued over five epochs. A batch size of 16 was used, and training continued for a maximum of 100 epochs with early stopping triggered after 10 epochs of non-improvement in validation loss. The final model was selected based on the best validation F1 score.

To ensure reproducibility and robust internal evaluation, five-fold cross-validation was employed. Each fold was constructed with strict patient-level separation and identical hyperparameter settings. All models were subsequently evaluated on an independent test cohort held out from training.

The computational environment consisted of high-performance hardware, including an Intel Core i9-10900X CPU, 128 GB of RAM, and two NVIDIA GeForce RTX A5000 GPUs with 24 GB of memory each. The code supporting this implementation is available for public access on Github, ensuring both transparency and reproducibility. This multi-view classification strategy, combined with the fine-tuned deep learning architecture, enhances the ability to accurately identify radiolucent foreign body aspirations on CT images. The internal modeling cohort for classification was trained and evaluated exclusively on radiolucent FBA and NFBA cases.

### Expert radiologists evaluation

To benchmark the model against expert radiologists’ performance, we conducted a blinded evaluation involving a panel of experienced thoracic radiologists. Specifically, three board-certified thoracic radiologists, each with over 10 years of clinical experience, independently reviewed all CT scans in the independent test cohort. All radiologists were blinded to model predictions, patient clinical history, and bronchoscopy results. Axial CT images were presented in a standalone DICOM viewer without additional contextual information.

Discrepancies among the three readers were resolved using a consensus protocol. In cases where two radiologists agreed and one disagreed, the majority vote determined the reference label. In the rare instances where all three readers provided divergent assessments, the case was jointly reviewed and resolved through discussion. These expert interpretations were used as the reference standard for performance comparisons with the deep learning model.

### Performance metrics

To comprehensively evaluate the performance of the classification model, we employed four standard metrics: accuracy, precision, recall, and F1 score. Accuracy measures the overall proportion of correctly predicted instances and provides a general sense of model performance. The F1 score serves as the harmonic mean of precision and recall, offering a balanced metric that is particularly useful when dealing with imbalanced datasets. Together, these metrics offer a comprehensive and reliable framework for evaluating classification performance. The formula is as follows:

2 $$P{recision}=\frac{{True\;Positives}}{{True}\,{Positives}+{False}\,{Positives}}$$

3 $${Recall}=\frac{T{rue\; Positives}}{T{rue\; Positive}+F{alse\; Negatives}}$$

4 $$F1{Score}=2\,\times \,\frac{P{recision}\times {Recall}}{P{recision}+{Recall}}$$

5 $$A{ccuracy}=\frac{{True\; Negatives}+{True\; Positives}}{{True\; Negatives}+{True\; Positives}+{False}\,{Negatives}+{False}\,{Positives}}$$

### Randomization and validation strategy

To ensure methodological rigor and prevent data leakage, we adopted a stratified five-fold cross-validation strategy with strict patient-level separation, such that no data from a single subject appeared in more than one fold. Stratified random sampling preserved the class distribution of radiolucent FBA and non-FBA cases across all folds, supporting stable learning and unbiased validation. A fixed random seed was used throughout the partitioning process to maintain reproducibility and enable consistent experimental conditions.

All preprocessing and data augmentation procedures were confined strictly to the training folds within each iteration, thereby eliminating the possibility of information leakage into the validation set. We elected not to implement chronological separation due to the narrow temporal span of data collection (2017–2024) and the low prevalence of radiolucent FBA, which would have severely limited the number of positive cases available for model training. Instead, the independent evaluation cohort and external validation dataset were entirely held out from the training pipeline and reserved for final model performance assessment, ensuring an unbiased evaluation of generalization capability.

### Statistical analysis

Continuous variables were assessed for normality using the Shapiro-Wilk test. Normally distributed data was expressed as mean (standard deviation) and compared between groups (FBA vs. NFBA) using the independent samples t-test. For non-normally distributed data, results were summarized as median (interquartile range, IQR) and compared using the Wilcoxon rank-sum test. Categorical variables were expressed as numbers (percentages) and compared using the chi-square test or Fisher’s exact test when the expected number of cells was less than 5. Two-sided *P* values less than 0.05 were considered statistically significant. All analyses were performed using R (version 4.4.2).

To compare model performance with expert radiologists interpretation on the independent evaluation dataset, McNemar’s test was used for paired sensitivity and specificity comparisons. Bootstrapping with 1000 iterations was used to estimate 95% confidence intervals and assess differences in F1 score. All analyses were performed in Python (v3.9) using SciPy and scikit-learn.

### Study design and ethical approval

This retrospective, multi-center study was conducted in compliance with the Standards for Reporting Diagnostic Accuracy Studies (STARD) guidelines^32^. Ethical approval was obtained from the institutional review boards of all participating centers: The Central Hospital of Wuhan (WHZXKYL2024-108), The Renmin Hospital of Wuhan University (WDRY2025-K083), and The Zhongnan Hospital of Wuhan University (2025086K). Due to the retrospective nature and full anonymization of imaging data, informed consent was waived. The study aimed to develop and evaluate a deep learning pipeline for detecting radiolucent foreign body aspiration from chest computed tomography scans by combining high-precision airway segmentation with multi-view convolutional classification. Three datasets were used: internal modeling, external validation, and independent evaluation, with strict cohort separation throughout all stages of development and testing.

## Supplementary information

Supplementary Information

## Data Availability

The data supporting the findings of this study are available upon reasonable request from the corresponding author with approval from the corresponding hospital. The source code for this implementation is publicly accessible at the following website: https://github.com/ZheChen1999/FBA_DL.
