# Supplementary material for: Automated detection of radiolucent foreign body aspiration on chest CT using deep learning
Source: NPJ Digit Med. 2025 Nov 10;8:647. doi: 10.1038/s41746-025-02097-w (PMC12603148; doi:10.1038/s41746-025-02097-w)
Supplement: Supplementary file 1 — Supplementary Information [file 41746_2025_2097_MOESM1_ESM.pdf]

# Supplementary Tables

**Table S1 | Patient Characteristics of Radiolucent FBA and NFBA in Internal Modelling and External Validation Cohorts.**

| | Internal Modelling (n=268) | | | External Validation (n=103) | | |
|---|---|---|---|---|---|---|
| | **FBA (n=41)** | **NFBA (n=227)** | ***P*-value** | **FBA (n=21)** | **NFBA (n=82)** | ***P*-value** |
| **Age, years** | | | | | | |
| | 66 (53-77) | 62 (53-77) | 0.04* | 66 (57-70) | 66 (57-70) | 0.67 |
| **Sex** | | | | | | |
| *Female* | 15 (36.6%) | 116 (51.1%) | 0.1232 | 8 (38.1%) | 44 (53.7%) | 0.3039 |
| *Male* | 26 (63.4%) | 111 (48.9%) | | 13 (61.9%) | 38 (46.3%) | |
| **BMI** | | | | | | |
| | 22.49 (20.76-24.51) | 22.83 (20.76-24.51) | 0.82 | 23.19 (20.83-25.1) | 23.07 (20.83-25.1) | 0.67 |
| **Length of disease course, days** | | | | | | |
| | 60 (10-120) | 7 (10-120) | <0.0001* | 60 (14-180) | 7 (14-180) | <0.0001* |
| **Hospital stays, days** | | | | | | |
| | 8 (6-10) | 7 (6-10) | 0.04* | 7 (5-9) | 7 (5-9) | 0.47 |
| **Experience in ICU** | | | | | | |
| *yes* | 9 (22%) | 12 (5%) | 0.0013* | 1 (4.8%) | 6 (7.3%) | 1 |
| *no* | 32 (78%) | 215 (95%) | | 20 (95.2%) | 76 (92.7%) | |
| **COPD** | | | | | | |
| *yes* | 8 (19.5%) | 35 (15.5%) | 0.6787 | 2 (9.5%) | 16 (19.5%) | 0.3536 |
| *no* | 33(80.5%) | 192 (84.5%) | | 19 (90.5%) | 66 (80.5%) | |
| **Asthma** | | | | | | |
| *yes* | 1 (2.4%) | 12 (5.3%) | 0.6987 | 3 (14.3%) | 6 (7.3%) | 0.3837 |
| *no* | 40 (97.6%) | 215 (94.7%) | | 18 (85.7%) | 76 (92.7%) | |
| **Bronchiectasis** | | | | | | |
| *yes* | 4 (9.8%) | 12 (5.3%) | 0.3634 | 1 (4.8%) | 11 (13.4%) | 0.4513 |
| *no* | 37 (90.2%) | 215 (94.7%) | | 20 (95.2%) | 71 (86.6%) | |
| **Lung cancer** | | | | | | |
| *yes* | 1 (2.4%) | 3 (1.3%) | 0.4888 | 0 (0%) | 3 (3.7%) | 1 |
| *no* | 40 (97.6%) | 224 (98.7%) | | 21 (100%) | 79 (96.3%) | |

| | | | | | | |
|---|---|---|---|---|---|---|
| **Interstitial lung disease** | | | | | | |
| *yes* | 1 (2.4%) | 6 (2.7%) | 1 | 2 (9.5%) | 3 (3.7%) | 0.2687 |
| *no* | 40 (97.60) | 221 (97.3%) | | 19 (90.5%) | 79 (96.3%) | |
| **Nervous system disease** | | | | | | |
| *yes* | 7 (17.1%) | 17 (7.5%) | 0.0697 | 2 (9.5%) | 7 (8.5%) | 1 |
| *no* | 34 (82.9%) | 210 (92.5%) | | 19 (90.5%) | 75 (91.5%) | |
| **Smoking history** | | | | | | |
| *yes* | 19 (46.3%) | 170 (74.9%) | 0.0084* | 7 (33.3%) | 22 (27%) | 0.7494 |
| *no* | 22 (53.7%) | 57 (25.2%) | | 14 (66.7%) | 60 (73.2%) | |
| **Pneumonic patch** | | | | | | |
| yes | 36 (87.8%) | 217 (95.6%) | 0.0616 | 8 (38.1%) | 79 (96.3%) | <0.0001* |
| no | 5 (12.2%) | 10 (4.4%) | | 13 (61.9%) | 3 (3.7%) | |
| **Airway stenosis** | | | | | | |
| *yes* | 6 (14.8%) | 17 (7.5%) | 0.1377 | 5 (23.8%) | 4 (4.9%) | 0.0163* |
| *no* | 35 (85.4%) | 210 (92.5%) | | 16 (76.2%) | 78 (95.1%) | |
| **Atelectasis** | | | | | | |
| *yes* | 19 (46.3%) | 41 (18.1%) | 0.0002* | 3 (14.3%) | 19 (23.2%) | 0.5526 |
| *no* | 22 (53.7%) | 186 (81.9%) | | 18 (85.7%) | 63 (76.8%) | |
| **Pleural effusion** | | | | | | |
| *yes* | 0 (0%) | 36 (15.9%) | 0.0125* | 1 (4.8%) | 14 (17.1%) | 0.2954 |
| *no* | 41 (100%) | 191 (84.1) | | 20 (95.2%) | 68 (82.9%) | |
| **Consolidation** | | | | | | |
| *yes* | 4 (9.8%) | 27 (11.9%) | 1 | 3 (14.3%) | 18 (22%) | 0.5539 |
| *no* | 37 (90.2%) | 200 (88.1%) | | 18 (85.7%) | 64 (78%) | |
| **Pulmonary emphysema** | | | | | | |
| *yes* | 4 (9.8%) | 62 (27.4%) | 0.0266* | 6 (28.6%) | 20 (24.4%) | 0.9108 |
| *no* | 37 (90.2%) | 165 (72.6%) | | 15 (71.4%) | 62 (75.6%) | |
| **Tuberculosis** | | | | | | |
| *yes* | 2 (4.9%) | 15 (6.6%) | 1 | 6 (28.6%) | 7 (8.5%) | 0.0236* |
| *no* | 39 (95.1%) | 212 (93.4%) | | 15 (71.4%) | 75 (91.5%) | |
| **Lung nodule** | | | | | | |
| *yes* | 11 (26.8%) | 130 (42.5%) | <0.0001* | 8 (38.1%) | 50 (61%) | 0.1011 |
| *no* | 30 (73.2%) | 97 (57.5%) | | 13 (61.9%) | 32 (39%) | |

Data are median (IQR) or n (%). *$P$-value < 0.05 with statistical significance
FBA: foreign body aspiration; NFBA: Non-foreign body aspiration.

**Table S2 | Radiolucent Foreign Body Characteristics in the Internal Modelling and External Validation Cohorts.**

| | Internal Modeling (n = 41) | External Validation (n = 21) | ***P*-value** |
|---|---|---|---|
| **Site of foreign body** | | | |
| *Glottis* | 0 (0%) | 0 (0%) | 0.1253 |
| *Windpipe* | 0 (0%) | 0 (0%) | |
| *Left main bronchus* | 3 (7%) | 0 (0%) | |
| *Left upper lobe bronchus* | 4 (10%) | 1 (5%) | |
| *Left lower lobe bronchus* | 5 (12%) | 2 (10%) | |
| *Right main bronchus* | 2 (5%) | 1 (5%) | |
| *Right upper lobe bronchus* | 11 (27%) | 0 (0%) | |
| *Right middle lobe bronchus* | 13 (32%) | 7 (33%) | |
| *Right lower lobe bronchus* | 1 (2%) | 10 (48%) | |
| *Multiple sites* | 2 (5%) | 0 (0%) | |
| **Type of foreign body** | | | |
| *Bone* | 15 (37%) | 8 (38%) | 0.2485 |
| *Medicine pill* | 1 (2%) | 0 (0%) | |
| *Plants* | 11 (27%) | 1 (5%) | |
| *Inorganics*# | 2 (5%) | 0 (0%) | |
| *Unknown* | 12 (29%) | 12 (57%) | |

Data are median (IQR) or n (%). **P*-value < 0.05 with statistical significance.
#Metal subjects, plastic films and dentures are classified as inorganics, while bones, medicine pills and plants as organics.

**Table S3｜Backbone Architecture comparison in the Independent Evaluation Cohort for Radiolucent FBA Cases**

| Backbone | TP | FN | TN | FP | Accuracy | Precision | Recall | F1 Score |
|---|---|---|---|---|---|---|---|---|
| **ResNet-18** | 10 ( 14.3%) | 4 (5.7%) | 53 (75.7%) | 3 (4.3%) | 90.0% | 76.9% | 71.4% | 74.1% |
| **EfficientNet-B0** | 9 (12.9%) | 5 (7.1%) | 53 (75.7%) | 3 (4.3%) | 88.6% | 75.0% | 64.3% | 69.2% |
| **DenseNet-121** | 9 (12.9%) | 5 (7.1%) | 52 (74.3%) | 4 (5.7%) | 87.1% | 69.2% | 64.3% | 66.7% |
| **ViT-B/16** | 8 (11.4%) | 6 (8.6%) | 52 (74.3%) | 4 (5.7%) | 85.7% | 66.7% | 57.1% | 61.5% |
| **Swin-T (tiny)** | 9 (12.9%) | 5 (7.1%) | 51 (72.9%) | 5 (7.1%) | 85.7% | 64.3% | 64.3% | 64.3% |

Data are n (%).TP: True Positive; FP: False Positive; TN: True Negative; FN: False Negative.
Interpretation: The full pipeline achieved the highest performance across all evaluation metrics, validating the importance of each module. These findings suggest that structural modeling and comprehensive spatial coverage are critical for detecting subtle FBA-related changes.
Note: Values in parentheses represent the percentage relative to the full cohort. Percentages for TP/FN/TN/FP are calculated with denominator = n (FBA+NFBA cases).

**Table S4 │ Age-based Subgroup Performance Comparison in the Independent Evaluation Cohort**

| Age Group | TP | FN | TN | FP | Accuracy | Precision | Recall | F1 Score |
|---|---|---|---|---|---|---|---|---|
| **< 40 years (n = 12)** | 3 (25.0%) | 1 (8.3%) | 7 (58.3%) | 1 (8.3%) | 83.3% | 75.0% | 75.0% | 75.0% |
| **≥ 40 years (n = 58)** | 7 (12.1%) | 3 (5.2%) | 46 (79.3%) | 2 (3.4%) | 91.4% | 77.8% | 70.0% | 73.7% |

Data are n (%).TP: True Positive; FP: False Positive; TN: True Negative; FN: False Negative.
Note: Values in parentheses represent the percentage relative to the full cohort. Percentages for TP/FN/TN/FP are calculated with denominator = n (FBA+NFBA cases).
All *P* values were calculated using Fisher's exact test.

## Table S5 | An Ablation Study in the Independent Evaluation Cohort for Radiolucent FBA Cases

| Radiolucent FBA cohort (n=70) | TP | FN | TN | FP | Accuracy | Precision | Recall | F1 Score |
|---|---|---|---|---|---|---|---|---|
| **Baseline (Raw CT Only)** | 8 (11.4%) | 6 (8.6%) | 43 (61.4%) | 13(18.6%) | 72.9% | 38.1% | 57.1% | 45.7% |
| **+ With Segmentation Mask** | 9 (12.9%) | 5 (7.1%) | 45 (64.3%) | 11 (15.7%) | 77.1% | 45.0% | 64.3% | 52.9% |
| **+ Fewer Views (6 Views)** | 9 (12.9%) | 5 (7.1%) | 47 (67.1%) | 9 (12.9%) | 80.0% | 50.0% | 64.3% | 56.3% |
| **+ With Data Augmentation** | 10 (14.3%) | 4 (5.7%) | 49 (70.0%) | 7 (10.0%) | 84.3% | 58.8% | 71.4% | 64.5% |
| **Full Pipeline (Proposed 12 Views)** | 10 ( 14.3%) | 4 (5.7%) | 53 (75.7%) | 3 (4.3%) | 90.0% | 76.9% | 71.4% | 74.1% |

Data are n (%).TP: True Positive; FP: False Positive; TN: True Negative; FN: False Negative.
Interpretation: The full pipeline achieved the highest performance across all evaluation metrics, validating the importance of each module. These findings suggest that structural modeling and comprehensive spatial coverage are critical for detecting subtle FBA-related changes.
Note: Values in parentheses represent the percentage relative to the full cohort. Percentages for TP/FN/TN/FP are calculated with denominator = n (FBA+NFBA cases).

**Table S6 | Patient Characteristics of Radiolucent FBA and NFBA in the Independent Evaluation Cohort**

| | FBA (n = 14) | NFBA (n = 56) | *P*-value |
|---|---|---|---|
| **Age, years** | | | |
| | 56 (38-74) | 59 (24-76) | 0.7185 |
| **Sex** | | | |
| *Female* | 6 (42.8%) | 24 (42.8%) | 0.3293 |
| *Male* | 8 (57.2%) | 32 (57.2%) | |
| **BMI** | | | |
| | 22.59 (20.61-24.35) | 23.85 (20.85-25.68) | 0.95 |
| **Hospital stays, days** | | | |
| | 7 (1-27) | 7 (2-28) | 0.4055 |
| **Experience in ICU, days** | | | |
| *yes* | 2 (14.3%) | 1 (1.8%) | 0.5286 |
| *no* | 12 (85.7%) | 55 (98.2%) | |
| **COPD** | | | |
| *yes* | 1 (7.1%) | 11 (19.6%) | 0.0915 |
| *no* | 13 (92.9%) | 45 (80.4%) | |
| **Asthma** | | | |
| *yes* | 0 (0%) | 7 (12.5%) | 1 |
| *no* | 14 (100%) | 49 (87.5%) | |
| **Bronchiectasis** | | | |
| *yes* | 1 (7.1%) | 7 (12.5%) | 0.5369 |
| *no* | 13 (92.9) | 49 (87.5%) | |
| **Interstitial lung disease** | | | |
| *yes* | 0 (0%) | 2 (3.6%) | 1 |
| *no* | 14 (0%) | 54 (96.4%) | |

Data are median (IQR) or n (%). **P*-value < 0.05 with statistical significance
FBA: foreign body aspiration; NFBA: Non-foreign body aspiration.
